# Successful Subretinal Delivery and Monitoring of MicroBeads in Mice

**DOI:** 10.1371/journal.pone.0055173

**Published:** 2013-01-28

**Authors:** M. Dominik Fischer, Tobias Goldmann, Christine Wallrapp, Regine Mühlfriedel, Susanne C. Beck, Gabi Stern-Schneider, Marius Ueffing, Uwe Wolfrum, Mathias W. Seeliger

**Affiliations:** 1 University Eye Hospital, Centre for Ophthalmology, University of Tübingen, Tübingen, Germany; 2 Nuffield Laboratory of Ophthalmology, University of Oxford, Oxford, United Kingdom; 3 Department of Cell and Matrix Biology, Institute of Zoology, Johannes Gutenberg University Mainz, Mainz, Germany; 4 CellMed AG, Alzenau, Germany; 5 Division of Ocular Neurodegeneration, University of Tübingen, Tübingen, Germany; 6 Institute for Ophthalmic Research, Centre for Ophthalmology, University of Tübingen, Tübingen, Germany; Schepens Eye Research Institute, Harvard Medical School, United States of America

## Abstract

**Background:**

To monitor viability of implanted genetically engineered and microencapsulated human stem cells (MicroBeads) in the mouse eye, and to study the impact of the beads and/or xenogenic cells on retinal integrity.

**Methodology/Principal Findings:**

MicroBeads were implanted into the subretinal space of SV126 wild type mice using an ab externo approach. Viability of microencapsulated cells was monitored by noninvasive retinal imaging (Spectralis™ HRA+OCT). Retinal integrity was also assessed with retinal imaging and upon the end of the study by light and electron microscopy. The implanted GFP-marked cells encapsulated in subretinal MicroBeads remained viable over a period of up to 4 months. Retinal integrity and viability appeared unaltered apart from the focal damage due to the surgical implantation, GFAP upregulation, and opsin mistargeting in the immediate surrounding tissue.

**Conclusions/Significance:**

The accessibility for routine surgery and its immune privileged state make the eye an ideal target for release system implants for therapeutic substances, including neurotrophic and anti-angiogenic compounds or protein based biosimilars. Microencapsulated human stem cells (MicroBeads) promise to overcome limitations inherent with single factor release systems, as they are able to produce physiologic combinations of bioactive compounds.

## Introduction

Hereditary retinal degenerative disorders (HRD) represent a genetically and phenotypically heterogeneous group of potentially blinding diseases, Retinitis Pigmentosa being the most prominent form [1]. By 2012, more then 200 retinal disease genes have been mapped and more than 150 identified (RetNet database: http://www.sph.uth.tmc.edu/Retnet/). HRD commonly affect the photoreceptors (rods and/or cones), the retinal pigment epithelium, and/or the processing and transmitting first and second order neurons. The considerable burden of visual disability and the tremendous socioeconomic impact in HRD are in strong contrast to the limited therapeutic options currently available. Recently, the first specific therapeutic strategies have now reached clinical phase I and II trial status in patients with RPE65-deficiency [2], [3]. Though very promising for some forms of HRD, the main disadvantage of corrective gene therapy is its narrow applicability due to its limitation to only one gene/mutation (also in terms of the regulatory approval procedure). However, recent work has provided evidence for the applicability of gene therapy even in dominantly inherited gain of function mutations [4]. In contrast, neuroprotective therapeutic strategies with a broader spectrum appear ideally suited to overcome this limited applicability and may help to prevent, retard, or reverse neuronal cell death in HRD regardless of its genetic and/or environmental causes [5], [6]. Over the last two decades, numerous neuroprotective factors have been tested for their therapeutic potential in a variety of HRD animal models [7], [8]. Among these, ciliary neurotrophic factor (CNTF) has shown promising results in its effect to rescue photoreceptors in several rodent and large animal models [5], [9], [10]. Sustained delivery systems have been designed in form of encapsulated cell technology (ECT), which allows for the continuous release of CNTF into the vitreous [11]. This compound has been evaluated in clinical phase II/III trials (http://clinicaltrials.gov/ct2/results?term=CNTF) e.g. in patients with early and late stage HRD [12]. As cell-based delivery of bioactive molecules may not be only limited to neuroprotective compounds but also molecules counteracting angiogenesis or inflammation, this delivery strategy may also be suited for sustained delivery of therapeutic molecules treating AMD or recurrent uveitis. We explored the potential of MicroBeads as potential intraocular delivery system with sustained release of putative therapeutic biologicals. Our research questions regarded the viability of the genetically engineered and microencapsulated human mesenchymal stem cells (MicroBeads), which was monitored by detecting eGFP expression as reporter protein. Furthermore, we monitored ocular and retinal integrity at multiple time points after implanting the MicroBeads in the mouse eye to test for severe adverse events.

## Methods

### Animals

In this experimental study, wild type (SV126) mice were used for implantation of MicroBeads with eGFP expressing mesenchymal stem cells into the subretinal space (n = 14). Animals were kept under standard laboratory conditions (25°C, 60% humidity and 12 h light (200 lux)/dark circle) with free access to animal food and water. The conditions of housing and experiments were in accordance with the ARVO Statement for the Use of Animals in Ophthalmic and Vision Research.

### Treatment

**Figure 1:**
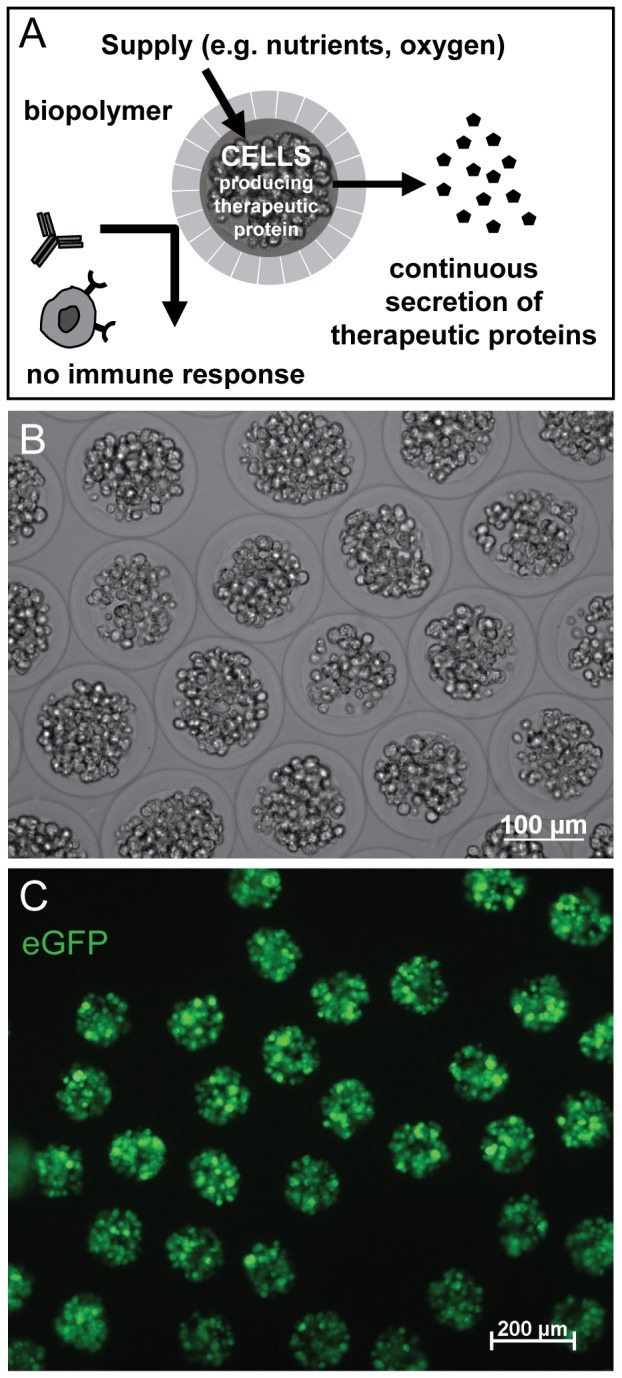
MicroBead characteristics. MicroBeads are alginate spheres with a diameter about 180 µm. (A) They may be used for a sustained release of therapeutic proteins while the producing cell population is protected from humoral and cellular immune response mechanisms. (B) For this study, beads with human mesenchymal stem cells were used that (C) express eGFP as reporter protein, which allowed to monitor localization, distribution and viability of MicroBeads *in vivo*.

MicroBeads are miniaturized alginate spheres with a diameter about 180 µm [13], which were provided by CellMed AG and can be used for a sustained release of therapeutic proteins while the producing cell population is protected from humoral and cellular immune response mechanisms (Fig. 1) [14]–[19]. Each MicroBead incorporates ca. 70 individual cells. These cells have been genetically modified to assure stable expression of eGFP. The cell line is of clonal origin and uniformly expresses eGFP in all cells. The parental human stromal (mesenchymal) stem cell was isolated by plastic adherence from a bone marrow aspirates of a 33 year old, healthy male donor and immortalized with the catalytic subunit of human telomerase reverse transcriptase gene (hTERT) [20]–[23]. The hMSC-TERT cell line was transfected with a plasmid expression vector encoding eGFP and selected with G418 for stable integration. After repeated single cell cloning a stable eGFP expressing cell clone was isolated. The same cell line was previously used in other published work using a very similar sustained delivery product, CellBeads®, which essentially only differs from MicroBeads by the size of the spherule [14], [18], [19], [24], [25].

The MicroBead matrix consists of alginate, a high molecular weight polysaccharide. The alginate network, which encapsulates the cells provides a pronounced diffusion barrier only for molecules >300 kDa. Over time the alginate is cleaved by spontaneous hydrolysis into smaller fragments, which cannot be bound to the hydrogel network anymore. These freely diffusible alginate fragments are finally cleared by the kidneys. Nevertheless the process of spontaneous hydrolysis is slow and will take longer than a year [22], [23]. The cellular debris is assumed to be cleaned by macrophages.

Mice were anesthetized by subcutaneous injection of ketamine (66.7 mg/kg) and xylazine (11.7 mg/kg). Pupillary mydriasis was achieved using topical tropicamide eye drops (Mydriaticum Stulln, Pharma Stulln GmbH, Stulln, Germany). Subretinal injections in SV126 mice were performed at age P28 using ≤10 µl of PBS solution. We injected MicroBeads expressing eGFP using an *ab externo* approach. Briefly, 26-gauge needles on 1 ml insulin syringes (Becton Dickinson, Heidelberg, Germany) were used for injections, which we performed under direct visualization using an operating microscope (Zeiss, Germany). Due to the 180 µm spherical dimension of the MicroBeads, the surgical implantation procedure had to be done with an instrument with >200 µm inner diameter (26G needles feature an inner/outer diameter of 260/464 µm). This caused considerable reflux and up to 10 µl had to be used to place up to 12 MicroBeads in the subretinal space. Care was taken to induce as little retinal detachment as possible. In all cases ca. 15–30% of the total retinal area was detached initially and spontaneously re-attached in all cases. A total of 14 mice underwent surgery. In 13 mice, MicroBeads could reliably be detected in the subretinal space after completion of surgical procedure. We suspect that the MicroBead in the remaining animal refluxed through the injection channel. Twelve MicroBeads were implanted in two animals, other animals received 4 MicroBeads (n = 1), 3 MicroBeads (n = 2), 2 MicroBeads (n = 2) or a single MicroBead (n = 6) in the subretinal space. A glass coverslip was placed over the cornea with a carbomer solution (Vidisic®, Dr. Mann Pharma, Berlin) as coupling agent to visualize the fundus for the injection. The tip was advanced through the sclera at the equator region into the subretinal space, and MicroBeads were injected under visual control into this compartment. The needle was removed slowly, and the animals recovered on a warming blanket. Postoperative anti-inflammatory and antibiotic treatment was provided for 48 h with Dexamethasone and Gentamicin ointment (Dexamytrex®, Bausch & Lomb, Berlin).

### Confocal scanning laser ophthalmoscopy (cSLO)

For evaluation of ocular health regarding cornea, anterior chamber, iris diaphragm, lens, vitreous and *en face* retinal imaging, we used the commercially available Heidelberg Engineering cSLO device HRA I (Heidelberg Engineering, Heidelberg, Germany) as described previously [26], [27]. Mice were subjected to cSLO imaging directly after the surgical implantation and at significant time points (10 d, 16 d, 42 d, 10 weeks and up to 4 months post surgery) thereafter to exclude postoperative complications and to evaluate localization, distribution and general viability of the MicroBeads by recording the eGFP signal using the autofluorescence mode of the cSLO (AF, λ 488 nm with barrier filter at 500 nm).

### Spectral Domain Optical Coherence Tomography (SD-OCT)

SD-OCT imaging was done in the same session as cSLO as previously described to control for structural integrity of mouse retina and MicroBeads [26], [28]. Briefly, line and volume scans were recorded as a mean of 16 images per B-Scan with automated alignment of iterative recordings using the Automated Real Time mode, thereby increasing the signal to noise ratio by a factor of four [29]. Resulting data were exported as 8 bit color bitmap files and processed in Adobe Photoshop CS3 (Adobe Systems, San Jose, CA).

### Light and electron microscopic analyses

Tissue preparation of retinal tissue for microscopic analyses was performed as previously described [30], [31]. Briefly, for fluorescence analyses, eyes were removed and either directly cryofixed in melting isopentane or fixed with 4% paraformaldehyde for 1 h, infiltrated in 30% sucrose and then cryofixed in melting isopentane. Cryosections (10 µm) were permeabilized, blocked and incubated with antibodies as described [30]. Fluorescence microscopy was performed with a Leica DM 6000 B (Leica microsystems, Bensheim, Germany). For transmission electron microscopy eyes were fixed in 2.5% glutaraldehyde in 0.1 M cacodylate buffer (pH 7.3) for 2 h. Specimens were washed and fixed in buffered 2% OsO_4_, dehydrated and embedded in araldite. Semi-thin (0.5 µm) and ultra-thin (60 nm) sections were cut on a Leica Ultracut S microtome. Ultrastructural analysis was performed using a Tecnai 12 BioTwin transmission electron microscope (FEI, Eindhoven, NL) and imaged with a SIS MegaView III SCCD camera. Images were processed with Adobe Photoshop CS.

### Antibodies, dyes& in vitro studies

The cocktail of monoclonal antibodies against opsin were previously described in Wolfrum and Schmitt [32]. Affinity-purified rabbit polyclonal antibodies against glial fibrillary acidic protein (GFAP) and vimentin were obtained from Dako (Glostrup, Denmark) and were used as alternative markers for glial stress in the retina [33]. Affinity-purified rabbit polyclonal antibody against vascular endothelial growth factor (VEGF) was obtained from Santa Cruz Biotech (Santa Cruz, USA). Secondary antibodies conjugated to Alexa 568 were from Molecular Probes (Leiden, The Netherlands). 4,6-Diamidino-2-phenylindole (DAPI; Sigma-Aldrich) was used for the visualization of nuclear DNA. For the in vitro studies, MicroBeads were kept at 5% CO_2_ and 37°C in 30 ml Eagle's minimal essential medium with 15% fetal calf serum and 2 mM L-Glutamine Sigma Aldrich (Schnelldorf, Germany). Medium was exchanged every 2–3 days.

## Results

**Figure 2:**
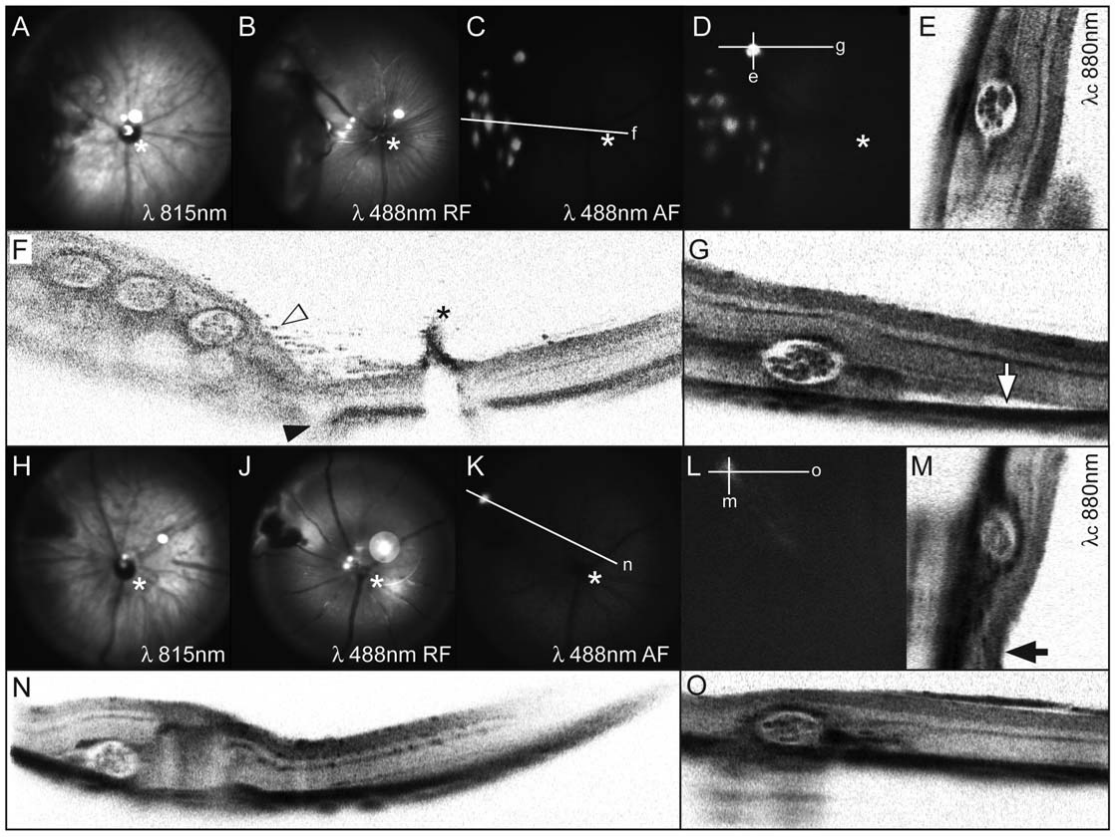
*In vivo* analyses after subretinal implantation of MicroBeads. Directly following implantation of 12 beads, (A) infrared and (B) red free cSLO imaging demonstrate the correct localization in the subretinal space and reveal a corresponding retinal detachment. (C) EGFP fluorescence of implanted MicroBeads as detected in autofluorescence mode. The white line indicates the orientation of the OCT virtual cross section (F) through the bead application site. The filled arrowhead indicates the position of the injection channel, and the empty arrowhead denotes dispersed retinal pigment epithelial cells (see explanation in text). The asterisk indicates the optic nerve head with Bergmeister papilla. The investigation of the same site ten days after surgery revealed that the eGFP signal remained strong (D), and corresponding virtual OCT cross sections (E,G) demonstrate continuing structural integrity of MicroBeads. Directly following injection of a single bead, the in vivo imaging revealed a more localized retinal detachment (H–K,N), which is completely resorbed ten days later (L–M,O).

**Figure 3:**
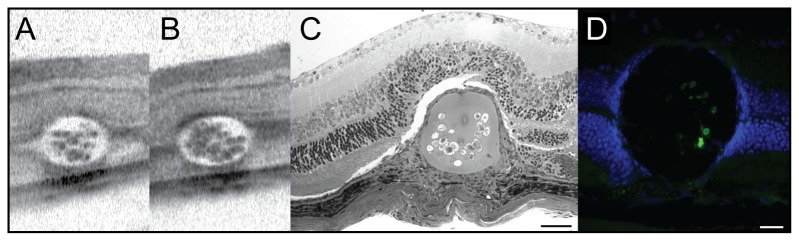
Comparison between *in vivo* and *in situ* analyses of subretinal beads. Noninvasive OCT virtual cross sections through MicroBeads allowed detection of single cells and demonstrate overall structural integrity of the sphere (A–B). Histology confirmed the subretinal location of the bead (C). Fluorescence microscopic analyses of the sphere allowed discerning eGFP signals of individual embedded cells (D). Blue: DAPI staining of retinal cell nuclei. Bars: C, 50 µm; D, 25 µm.

Mice were subjected to noninvasive imaging only 10 minutes after surgery in a timeline analysis protocol. This included *en face* cSLO imaging to analyze overall integrity of the eye and to document changes to corneal, lenticular, vitreal and retinal anatomy. The resolution of the noninvasive ocular imaging allowed detection of single cells in the MicroBeads (Fig. 2, 3). All surgically treated eyes maintained intact anterior segments and developed no epitheliopathy or cataract formation throughout the study. In some animals, we observed minimal retinal hemorrhages associated with the surgical procedure. However, all hemorrhages were self-limiting and had resolved spontaneously by the follow-up recordings 10 days after surgery. There were no serious adverse events due to the surgery and/or implantation of MicroBeads and all mice maintained good ocular and general health throughout the study.

In the initial experiments, several (up to 12) MicroBeads per eye were implanted into the subretinal space, which resulted in significant retinal detachment and disruption of retinal integrity (Fig. 2A–G). The *in vivo* quality control of our *ab externo* approach immediately following the implantation procedure using the infrared mode (IR, λ 815 nm) of the cSLO revealed defects at the level of the retinal pigment epithelium (RPE) (Fig. 2A,H). In particular, the injection channel was evident as was dispersion of pigmented cells into the vitreous (Fig. 2 F). While intravitreal placement of MicroBeads could be ruled out by funduscopy, cSLO and OCT imaging, small (self-sealing) retinal breaks might have caused RPE cell displacement. Using the red free mode (RF, λ 488 nm), retinal nerve fiber bundles made of axons from ganglion cells in the inner retinal layers could be analyzed, which appeared not to be affected by the procedure (Fig. 2B). Autofluorescence (AF) cSLO imaging showed eGFP as reporter protein in the MicroBeads (Fig. 2C). This facilitated both the efficient detection of MicroBeads and the long-term assessment of viable cells contained therein. SD-OCT allowed simultaneous en face detection and virtual cross sectioning of implanted beads with (Fig. 2F). After a period of ten days, all MicroBeads still produced strong eGFP signals (Fig. 2D) and maintained their regular structural appearance (Fig. 2E,G and. Fig. S2).

The implantation of single beads yielded similar results but with less structural damage (Fig. 2H–O). Locally, the implantation of both multiple and single MicroBeads resulted in altered retinal architecture with persistent focal retinal detachment as seen in Fig. 2G, or with localized degenerative changes as seen in Fig. 2M.

Subsequent *in situ* analyses corresponded fairly well with *in vivo* imaging results (Fig. 3) even though eGFP signal strength and number of eGFP positive encapsulated cells differed somewhat between in situ (Fig. 3D) and in vitro data (Fig. 1C). Additional experiments provided evidence that this difference in eGFP signal strength and number of eGFP positive encapsulated cells is not due to e.g. a rapid death of encapsulated cells upon intraocular delivery: MicroBeads from the same batch were either implanted as previously or kept *in vitro*. After 10 days, MicroBeads were imaged *in vivo* using cSLO or transilluminescence microscopy *in vitro* before both samples were processed using the same fixation/embedding methods and imaged by confocal microscopy (Fig. S1). These data suggest that tissue processing *ex vivo* (cryosectioning etc.) leads to artificial disintegration of alginate polymer material and subsequent loss of cells. In contrast, both in vivo SLO data as well as transilluminescence microscopy *in vitro* of the same batch of MicroBeads prior to tissue processing suggest robust eGFP expression after 10 days (Fig. S1, S2). Furthermore, data in Fig. 3 C–D and Fig. S2 demonstrate that due to the spherical shape and size of the Microbeads (180 µm), only a fraction of encapsulated cells can be expected in the plane of a histological (10 µm) section. Additionally, “micropositioning” of intraocular MicroBeads *in vivo* with variable transparency along the optical path are further reasons for differences in fluorescence signal derived with cSLO *in vivo* and using confocal microscopy on cryosections (Fig. S2).

**Figure 4:**
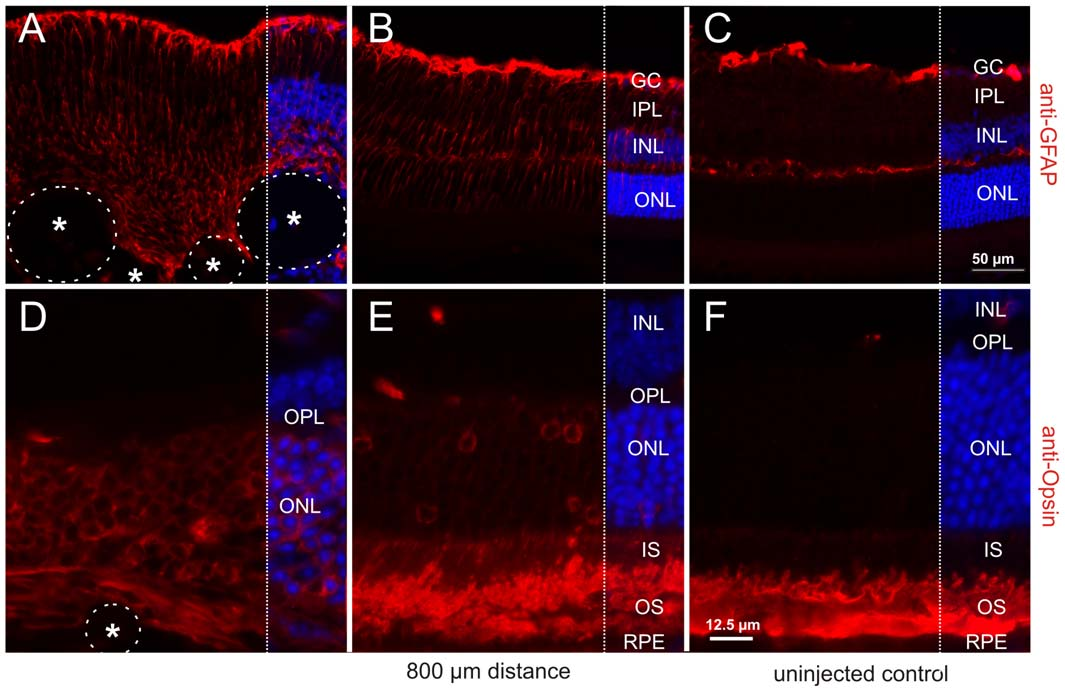
Focal damage following surgical implantation of MicroBeads. Indirect immunofluorescence of molecular markers for retinal degeneration was used to monitor side effects. Subretinal implantation was accompanied by GFAP upregulation (A–B) and opsin mislocalization to the inner segment (IS) and the outer nuclear layer (ONL) (D–E) at the site of MicroBead (asterisk) implantation and in a distance of 800 µm, which was not detected in uninjected controls. RPE: retinal pigment epithelium; OS: outer segment; OPL, outer plexiform layer; INL: inner nuclear layer; IPL: inner plexiform layer; GC: ganglion cells and Müller glia feat. Scale bars: 50 µm, 12.5 µm.

Immunohistochemical analysis of the retina directly adjacent to the subretinal MicroBeads revealed an upregulation of GFAP indicating Müller glia cell activation [34]. The intensity of the GFAP signal declined with increasing distance but remained slightly elevated within a radius of up to 800 µm around the site of implantation (Fig. 4A–C). Simultaneously, we observed opsin mislocalization in photoreceptor cells, which was most pronounced directly at the lesion site and again diminished as a function of distance with only minor opsin staining in the inner segments and outer nuclear layer at a distance of 800 µm (Fig. 4D–F).

**Figure 5:**
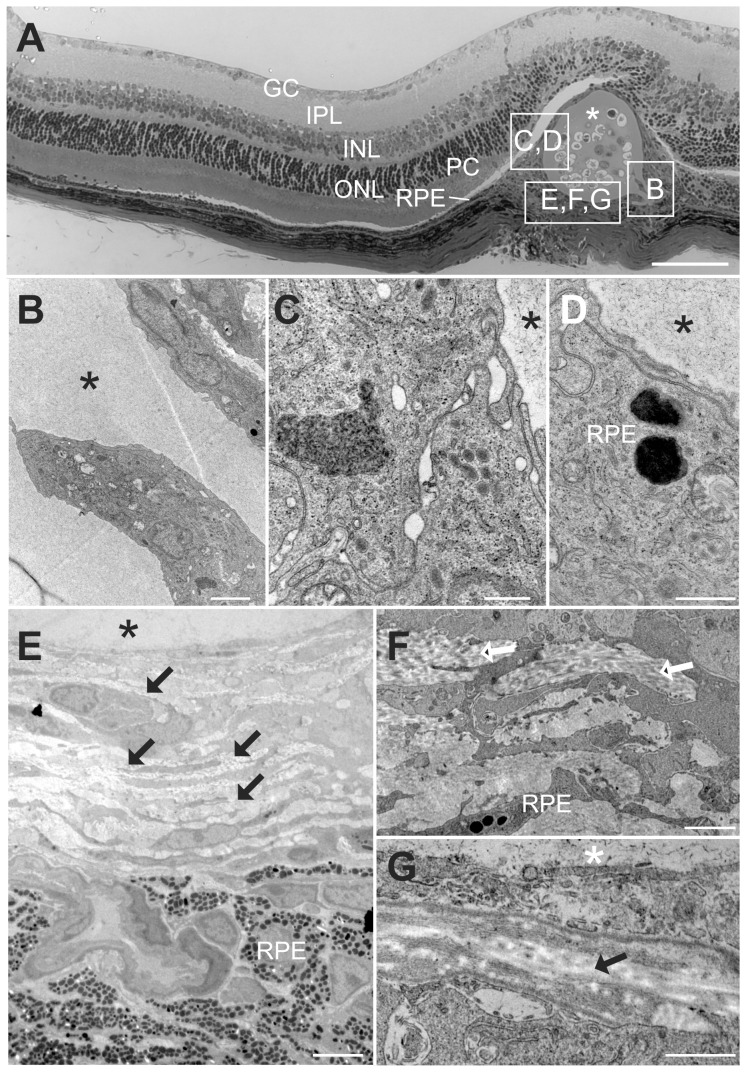
Ultrastructural changes of the retina in respect to subretinal injected MicroBeads. Ultrathin sections of subretinal injected MicroBeads were analyzed by transmission electron microscopy. (A) Overview of the area after subretinal MiroBead injection. Retinal integrity is altered at the site of implantation: Normal photoreceptor cell (PC) composition with outer and inner segments is not any longer visible. Furthermore, the outer (ONL) and the inner nuclear layer (INL) are fused and thereby the outer plexiform layer (OP) is not longer visible. The inner plexiform (IPL) and the ganglion cell layer (GC) do not show any gross morphological changes. (B–G) Higher magnifications of areas indicated in A. (B) Surface invagination into the alginate capsule of the MicroBead (asterisk). (C–G) Analyses of connective tissue and confluent retinal pigment epithelial cells (RPE), which closely engulfs the MicroBead (asterisk). Collagen fibrils (arrows) are visible in the RPE and connective tissue. Bars = 2.5 µm (A); 0.5 µm (B, C); 5 µm (D) 2 µm (E), 1 µm (F).

The ultra-structural analysis of subretinally placed MicroBeads permitted important insights into the interaction between the alginate surface and RPE cells (Fig. 5). Retinal pigment epithelial (RPE) cells closely interacted with the MicroBeads, and collagen fibers were apparent in the extracellular matrix of the confluent connective tissue adjacent to the alginate capsule. The interaction between RPE and MicroBeads displayed similarities to previously described observations on the active role of RPE cells in bone spicule formation in Retinitis pigmentosa [35]. In one instance, we observed surface invaginations into the alginate sphere (Fig. 5A, B). This stresses the need for a meticulous surgical procedure that minimizes any trauma to retina, RPE, and of course the MicroBeads themselves.

**Figure 6:**
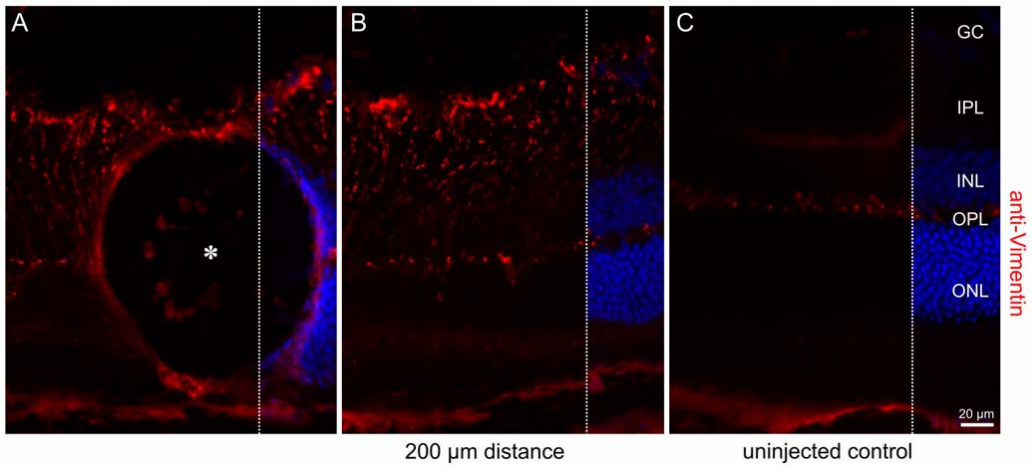
Retinal alterations ten weeks after surgical implantation of a MicroBead. Indirect immunofluorescence using anti-vimentin antibodies indicates vimentin upregulation at the site of the MicroBead implantation (A–B) compared to the uninjected control (C). ONL, outer nuclear layer; OPL, outer plexiform layer; INL: inner nuclear layer; IPL: inner plexiform layer; GC: ganglion cells and Müller glia feat. Scale bar: 20 µm.

**Figure 7:**
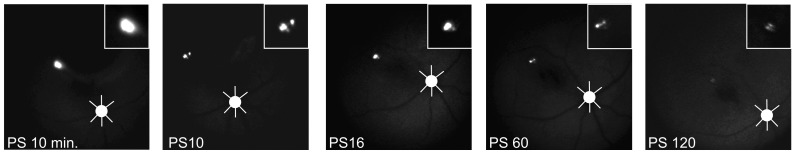
*In vivo* time line analysis of eGFP signal in one individual animal after subretinal implantation of two MicroBeads. Time points are indicated as 10 minutes post surgery (left) and as 10, 16, 60 and 120 days post surgery (PS). Individual location of the optic disc with its main vessels are indicated graphically for better orientation and insets highlight the eGFP fluorescence signal originating from the implanted MicroBeads.

Long-term integrity of MicroBeads was monitored *in vivo* by repetitive SLO autofluorescence imaging, and markers of inflammation studied immunohistochemically subsequently. For this, we investigated the immediate retinal area around subretinal MicroBeads ten weeks post-surgery using anti-vimentin, an alternative marker to anti-GFAP for Müller glia cell stress (Fig. 6 A–C). After ten weeks, MicroBead inplanted retinas still showed increased glial activity with upregulation of vimentin expression at the site of implantation (A–B) compared to an uninjected control (C). However, in contrast to the retina of retinal degeneration mouse models rd1 and rds we did not observe any evidence for an upregulation of VEGF in response to subretinal implantation of MicroBead in to the wildtype mouse retina (see Fig. S3). Long term follow up of eGFP reporter protein fluorescence using noninvasive cSLO imaging demonstrated decreasing signal strength over time. At P120, we saw remaining fluorescence of the MicroBeads in 3 of 5 animals. Background signal was elevated in one of the remaining eyes with 4 implanted MicroBeads, which prohibited longitudinal assessment of the fluorescence signal from MicroBeads. The other eye originally showed one fluorescent MicroBead, but at P120 no fluorescence was found although optical media were good and background was comparable to the contralateral untouched eye. Residual eGFP signal up to four months (the longest period tested) after the surgical procedure (Fig. 7, Fig. S4) indicates the potential of encapsulated cells for sustained delivery of biologicals.

## Discussion

The accessibility for routine surgery and its immune privileged state make the eye an ideal target for local interventional therapies minimizing if not avoiding systemic side effects and the necessity of considerable higher dosages of any putative therapeutic biological. Viral gene therapy is a prominent example. However, replacement or additive gene therapy is mutation or at least gene specific and the extensive genetic heterogeneity thus call for a more broadly applicable therapeutic approach. Neuroprotective strategies promise therapeutic efficacy through preventing or delaying the final common pathway of most retinal degenerative disorders - neuronal cell death by apoptosis. However, even intravitreal bolus injections of neuroprotective agents such as CNTF can cause severe side effects including retinal remodeling [36], [37]. Therefore sustained delivery systems that combine cell based delivery and recombinant expression of therapeutic agents after intravitreal implantation might have the potential to continuously provide therapeutic levels of neuroprotective as well as anti-angiogenic or anti-inflammatory compounds. Indeed, CellBeads®, a similar but larger vehicle compared to MicroBeads, was shown to provide sustained delivery of GLP-1 into the vitreous after intravitreal application in rats [15]. The same authors also report a neuroprotective effect of CellBeads® in the optic nerve crush model [18].

The current study was designed to determine the short and intermediate term effect of subretinal delivery of MicroBeads in the mouse eye with encapsulated cells producing eGFP as reporter construct. Our data underscore the applicability of such an approach in a retinal setting. Furthermore, the *in vivo* diagnostics used in this work open the possibility to perform time line analyses in individual treated animals to evaluate the treatment effect longitudinally. While intraocular implantation may induce a spatially limited glia cell response as indicated by GFAP/vimentin activity and other local changes, the MicroBeads remained stationary and functional throughout the four months investigated in the present study. Due to the nature of the MicroBead itself and the compounds to be released in future MicroBead systems, these are not intended to provide a lifelong cure following a single treatment. Rather, we see their role in symptomatic therapeutic strategies with an intermediate time frame. The current study was designed to determine the short and intermediate term effect of subretinal delivery of MicroBeads in the mouse eye with encapsulated cells producing eGFP as reporter construct. Additional long-term observations with potentially further improved MicroBeads compound materials will help to determine the performance of MicroBeads in the intraocular environment for periods of e.g. a year. Further, we will confirm that the time course of GFP expression observed approximately correlates with the ability of the encapsulated cells to secrete neuroprotective agents. One aspect important in this regard was the apparent epithelial-mesenchymal cell interaction (Fig. 5) after subretinal delivery. While intravitreal delivery might limit these interactions, the latter approach might render MicroBeads non-stationary. This would hinder the exact targeting of retinal structures and thus the therapeutic efficacy. Linked to this is another issue, the necessity to control and properly dose the production and secretion of therapeutic molecules, as an overdose may be toxic. As such, regulatory gene expression using inducible promoters to drive transgene expression may be needed in the future to ensure not only a long-lasting but also a constant treatment effect at safe therapeutic levels. Additionally, inclusion of a cellular suicide gene such as thymidine kinase may be desirable to secure against dysplastic transformation or uncontrolled proliferation of the mesenchymal cells.

Our data demonstrate a slow decline of eGFP derived signal strength over time *in vivo* (Fig. 7 and Fig. S4). Differences in eGFP signal strength and number of eGFP positive encapsulated cells between *in vitro* and *in vivo* data (Fig. 1C, Fig. 2, 3A–B and Figures S2 and S3A) and histological sections from tissue 10 days post surgery (Fig. 3C–D and Fig. S3B) is mostly due to the different imaging methodologies used and not due to a burst of cell loss after surgical intervention. Indeed, Fig. S1 and S2 demonstrate how histological processing affects eGFP signal strength and/or numbers of encapsulated cells. To eliminate methodological variance, we therefore assessed eGFP signal strength on iterative recordings with noninvasive retinal imaging in individual animals (Fig. 7) to monitor signal decay over time. However, this method does not allow precise quantitative comparisons. The main cause are factors that limit the amount of light on its path to and from the object, which include corneal and lens translucency (influenced by anesthesia), pupil width (influenced by dilating drug concentration), exact “micropositioning” in front of the cSLO and the fact that the cSLO recording system does not even have a scale on the intensity dial as the absolute values are arbitrary. Since all of these factors apply to non-fluorescent neighboring tissue areas, too, a qualitative assessment does however make sense.

Hence, despite these limitations, this study showed the potential of MicroBeads as intraocular delivery system as eGFP signal could be observed up to 4 months after implantation *in vivo*. The strong eGFP signal that we observed during this period indicates the potential of MicroBeads to continuously translate the engineered target protein. We conclude that microencapsulated human mesenchymal stem cells packed into MicroBeads show promise to overcome current limitations of compound delivery, as they are capable of reside viable and functional within the eye for a considerable period of time.

Finally, even though MicroBeads are essentially miniaturized CellBeads® specifically engineered for the use in the mouse eye, size might still be a critical issue and one can reasonably expect better results in a larger animal model. Even though retinal architecture and dimensions of total retinal thickness are similar in small and larger animal models, volume of vitreous, distance between retina and posterior lenticular capsule and area of retinal tissue are greater in larger animal models. This would make such a surgical approach much easier and large implanted devices have shown to be well tolerated in the subretinal space of larger animal models e.g. in case of the subretinal retinal prosthetic device [24], [25]. However, the availability of specific murine models of particular types of retinal degeneration is a strong argument for using mice when exploring the therapeutic spectrum. These well-characterized disease models are readily available for above-mentioned future experiments on the neuroprotective efficacy of MicroBead based sustained delivery of biologicals. Also, using these common disease models would allow to benchmark any putative therapeutic effect with results in the literature that were achieved by any other means. Hence, the benefits may outweigh the drawbacks of using mice in these experiments. Further experiments will also have to investigate the microenvironment after implantation such as host-implant interaction, opsin mislocalization and Muller cell activation. It will be of paramount importance to include relevant controls when testing the therapeutic potential of MicroBeads with hMSCs specifically designed to secrete e.g. neurotrophic factors.

In summary, we show here that cells encapsulated in alginate-based MicroBeads survived subretinally and continuously expressed eGFP over a period of at least 4 months, indicating their potential for sustained delivery of biologicals and making them a promising candidate for intermediate-term therapeutic use in human HRD patients.

## Supporting Information

Figure S1 **Processing of MicroBeads** ***in vitro*** **.** (A) Bright field (left) and fluorescence (right) microscopy of MicroBeads at day 0 (d0), when MicroBeads from the identical batch were implanted *in vivo* (Fig. S2). (B) Iterative recording of MicroBeads *in vitro* at day 10 (d10), when cSLO recording and tissue processing was performed in the animal in parallel (Fig. S2). (C–E) MicroBeads are recorded at various stages of tissue processing towards confocal microscopy. MicroBeads after 1 h fixation in 4% PFA and sucrose dehydration (C) still feature similar structure and fluorescence pattern. Thin (10 µm) cryosection of the 180 µm thick MicroBeads demonstrates four different levels of sectioning with the largest diameter holding ca. 15 cells, two holding 3–4 cells and one holding only one cell. All cells are eGFP positive and some appear outside of any alginate capsule, possibly indicating artificial displacement by sectioning. (E) Washing/permeabilization of sections and mounting slides for confocal microscopy seemingly dissolves the alginate polymer and leaves cells without structural support and ready for further displacement and loss in the process. All remaining cells are still eGFP positive.
(TIF)

Figure S2 **Confocal image of cryosection and** ***in vivo*** **cSLO imaging data 10 days post surgery.** Top panel shows 10 µm thick cryosection with a MicroBead containing eGFP positive cells in close proximity of the injection site (asterisk). *In vivo* autofluorescence at the same site of this individual animal (bottom) shows far more eGFP positive cells only minutes before tissue dissection and processing for histology. Scale bar: 50 µm.
(TIF)

Figure S3 **Indirect immunofluorescence revealed no induction of VEGF in response to subretinal MicroBead implantation.** Indirect immunofluorescence of anti-VEGF of retinal cryosections at the site of MicroBead implantation (A) in 800 µm distance of the implantation -site (B) and of un-implanted control mouse. Indirect immunofluorescence of anti-VEGF of rd1 (D) and rds (E) mouse retinas. In contrast to rd and rds mouse retinas, VEGF expression was not increased after subretinal MicroBead implantation. ONL, outer nuclear layer; OPL, outer plexiform layer; INL: inner nuclear layer; IPL: inner plexiform layer; GC: ganglion cells and Müller glia feat. Scale bar: 20 µm.
(TIF)

Figure S4 ***En face*** **and virtual cross sections of MicroBeads 120 days post surgery (PS).** Individual location of the optic disc with its main vessels are indicated graphically for better orientation and arrows highlight the eGFP fluorescence signal originating from the implanted MicroBeads.
(TIF)
