# Supplementary figures and images for: Successful Subretinal Delivery and Monitoring of MicroBeads in Mice
Source: PLoS One. 2013 Jan 28;8(1):e55173. doi: 10.1371/journal.pone.0055173 (PMC3557268; doi:10.1371/journal.pone.0055173)

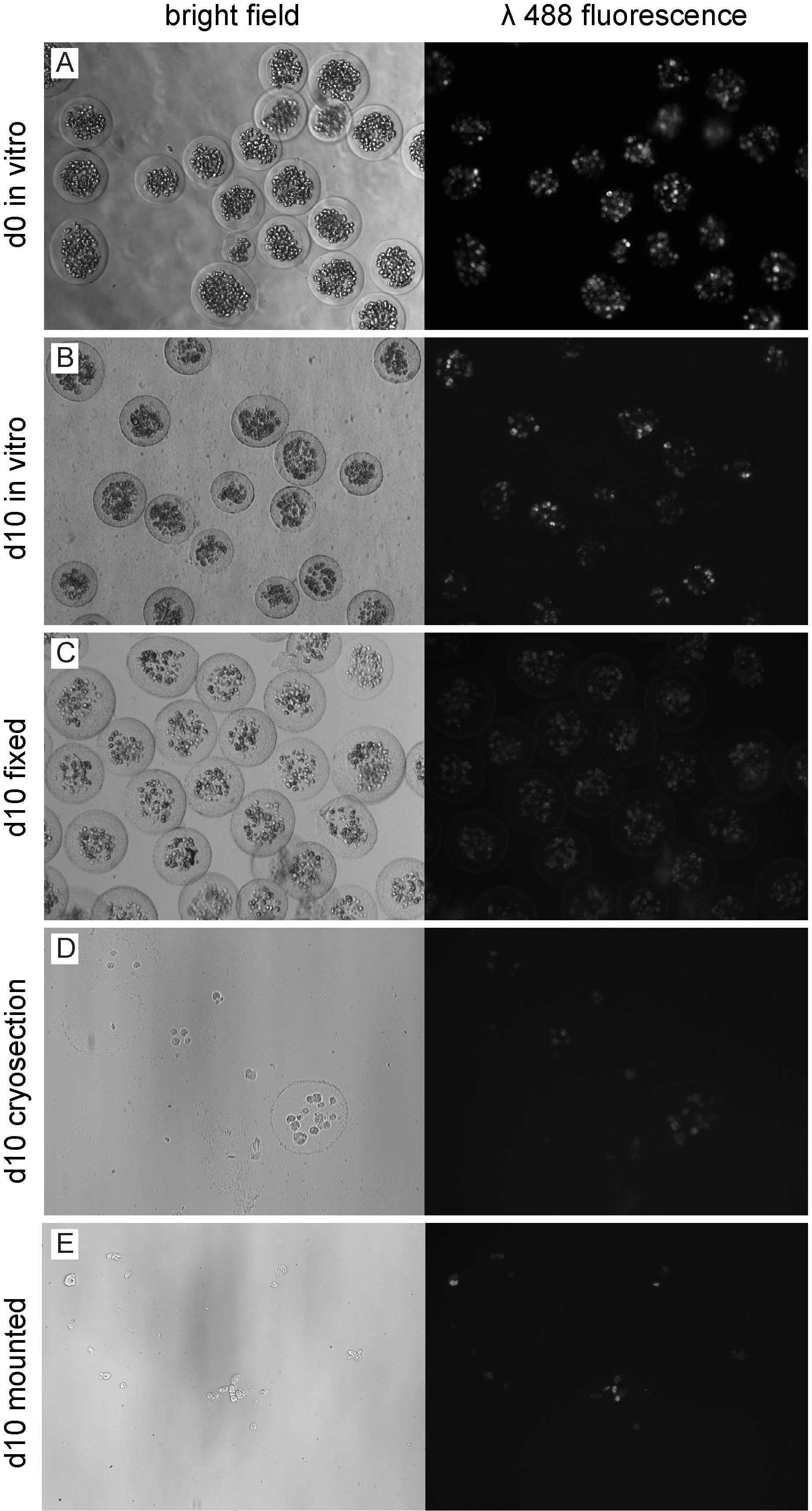

Supplement: Figure S1 — Processing of MicroBeads in vitro . (A) Bright field (left) and fluorescence (right) microscopy of MicroBeads at day 0 (d0), when MicroBeads from the identical batch were implanted in vivo (Fig. S2). (B) Iterative recording of MicroBeads in vitro at day 10 (d10), when cSLO recording and tissue processing was performed in the animal in parallel (Fig. S2). (C–E) MicroBeads are recorded at various stages of tissue processing towards confocal microscopy. MicroBeads after 1 h fixation in 4% PFA and sucrose dehydration (C) still feature similar structure and fluorescence pattern. Thin (10 µm) cryosection of the 180 µm thick MicroBeads demonstrates four different levels of sectioning with the largest diameter holding ca. 15 cells, two holding 3–4 cells and one holding only one cell. All cells are eGFP positive and some appear outside of any alginate capsule, possibly indicating artificial displacement by sectioning. (E) Washing/permeabilization of sections and mounting slides for confocal microscopy seemingly dissolves the alginate polymer and leaves cells without structural support and ready for further displacement and loss in the process. All remaining cells are still eGFP positive. (TIF) [file pone.0055173.s001.tif]

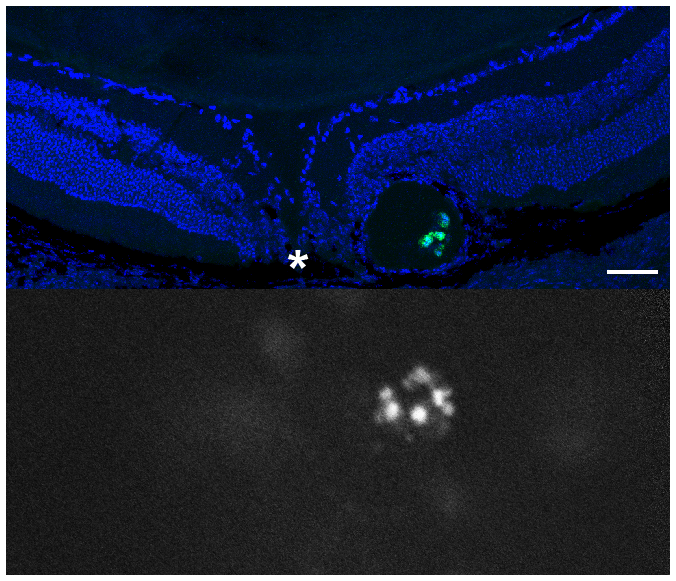

Supplement: Figure S2 — Confocal image of cryosection and in vivo cSLO imaging data 10 days post surgery. Top panel shows 10 µm thick cryosection with a MicroBead containing eGFP positive cells in close proximity of the injection site (asterisk). In vivo autofluorescence at the same site of this individual animal (bottom) shows far more eGFP positive cells only minutes before tissue dissection and processing for histology. Scale bar: 50 µm. (TIF) [file pone.0055173.s002.tif]

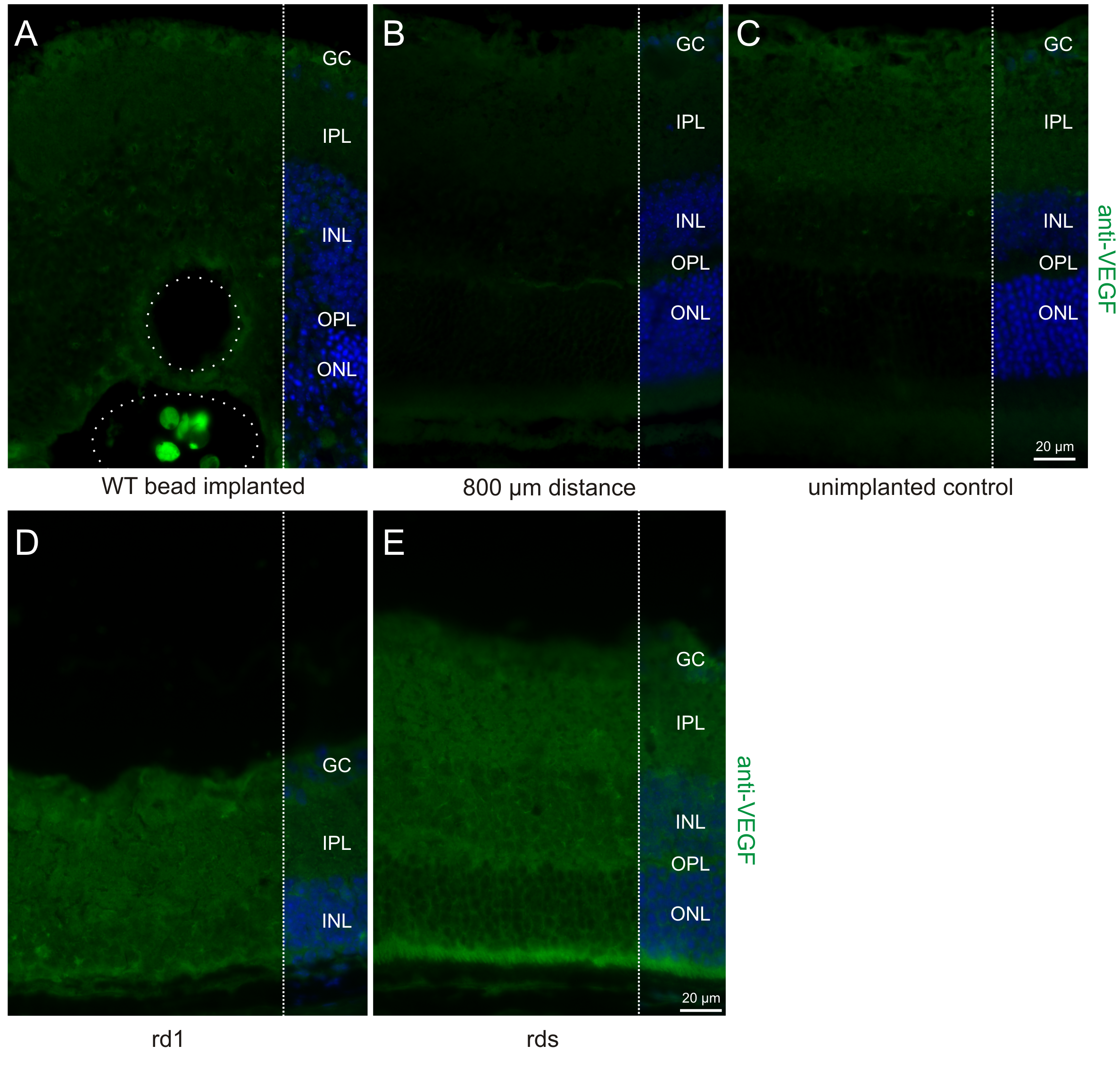

Supplement: Figure S3 — Indirect immunofluorescence revealed no induction of VEGF in response to subretinal MicroBead implantation. Indirect immunofluorescence of anti-VEGF of retinal cryosections at the site of MicroBead implantation (A) in 800 µm distance of the implantation -site (B) and of un-implanted control mouse. Indirect immunofluorescence of anti-VEGF of rd1 (D) and rds (E) mouse retinas. In contrast to rd and rds mouse retinas, VEGF expression was not increased after subretinal MicroBead implantation. ONL, outer nuclear layer; OPL, outer plexiform layer; INL: inner nuclear layer; IPL: inner plexiform layer; GC: ganglion cells and Müller glia feat. Scale bar: 20 µm. (TIF) [file pone.0055173.s003.tif]

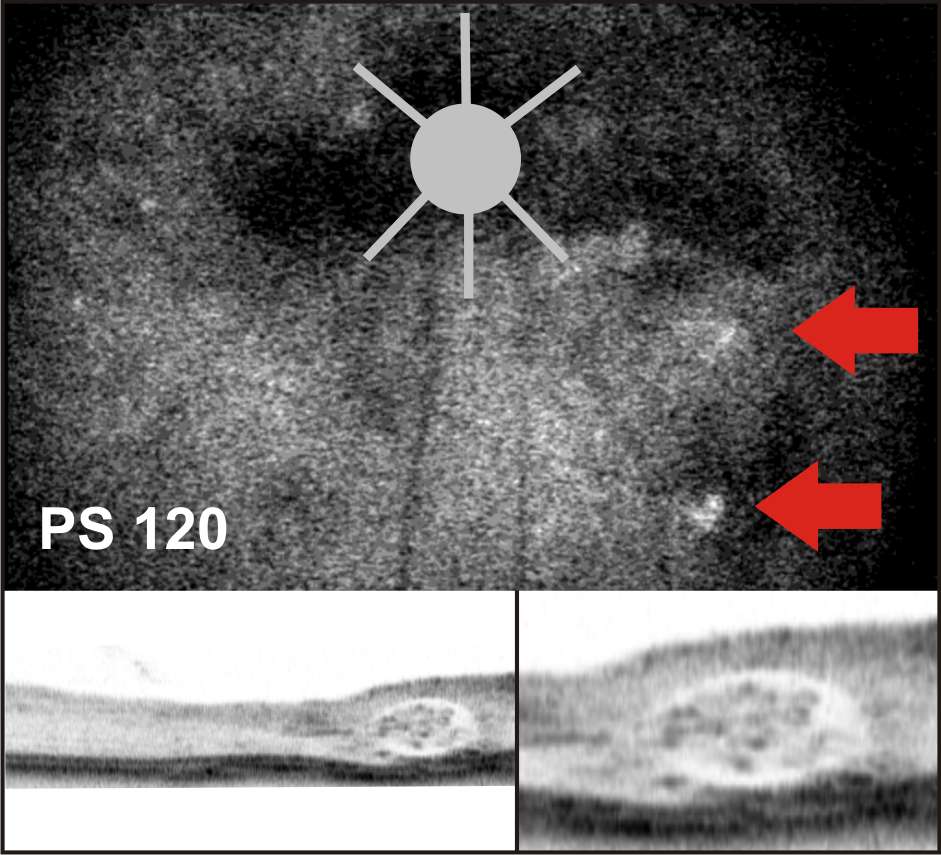

Supplement: Figure S4 — En face and virtual cross sections of MicroBeads 120 days post surgery (PS). Individual location of the optic disc with its main vessels are indicated graphically for better orientation and arrows highlight the eGFP fluorescence signal originating from the implanted MicroBeads. (TIF) [file pone.0055173.s004.tif]
